# Structural Repair of Reduced Graphene Oxide Promoted by Single‐Layer Graphene

**DOI:** 10.1002/advs.202410088

**Published:** 2024-12-30

**Authors:** Minghao Guo, Hong Yuan, Kun Ni, Chuanren Ye, Fei Pan, Juan Xiong, Yanwu Zhu

**Affiliations:** ^1^ Hefei National Research Center for Physical Sciences at the Microscale Department of Materials Science and Engineering School of Chemistry and Materials Science Key Laboratory of Precision and Intelligent Chemistry University of Science and Technology of China Hefei Anhui 230026 China

**Keywords:** graphene, graphitization, reduced graphene oxide, structural repair

## Abstract

High‐temperature graphitization of graphene oxide (GO) is a crucial step for enhancing interlayer stacking and repairing the in‐plane defects of reduced graphene oxide (rGO) films. However, the fine control of the structural repair and reducing the energy consumption in thermal treatment remain challenges. In this study, *ab‐initio* molecular dynamics simulations combined with experiments are used to investigate the structural evolution of rGO upon thermal annealing, with or without the presence of single‐layer graphene (SLG). It is found that the SLG promotes the repair of the carbon honeycombed matrix and alleviates the release of carbon‐containing groups during graphitization. Further electronic analysis reveals the crucial role of charge transfer from SLG to defective graphene in strengthening the C─C bonds. The result of Raman spectroscopy matches with the simulation, demonstrating an improved repairing degree of rGO upon contacting with SLG. This study provides a deeper understanding of the graphitization and repair mechanism of rGO, which may be useful for the highly efficient preparation of high‐quality graphene films.

## Graphical abstract

Single‐layer graphene (SLG) is utilized to promote the reduction of graphene oxide (GO) for fewer carbon losses and better structural restoration. The carbon skeleton is stabilized by the enhanced carbon‐carbon bonding strength, which originated from the electron transfer from SLG to GO.

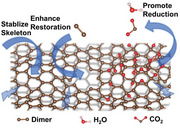

## 1 Introduction

With high thermal conductivity (≈2000 W·m^−1^·K^−1^),^[^ ^1^ ^]^ low thermal expansion coefficient (≈−8.0 × 10^−6^ K^−1^),^[^ ^2^ ^]^ superior carrier mobility (≈1.5 × 10^4^ cm^2^ V^−1^ s^−1^),^[^ ^3^ ^]^ high mechanical modulus (≈130 GPa) ^[^ ^4^ ^]^ and excellent chemical stability, graphene has emerged for applications in thermal management,^[^ ^1^ ^]^ electronic devices,^[^ ^5^ ^]^ energy storage ^[^ ^6^ ^]^ and so on. Specifically, when graphene is stacked into highly ordered films for applications in heat dissipation or electrical conductors/antenna, the performance is significantly reliant on the structural integrity of graphene.^[^ ^7^ ^]^ So far, a well‐built protocol for preparing highly ordered graphene films includes dispersing graphene oxide (GO) platelets in water, then assembling platelets by removing water, and finally annealing the dried GO films at high temperatures.^[^ ^7b^ ^]^ In thermal annealing, the residual oxygen‐containing groups in graphene are nearly entirely removed at 1500 °C, and the structural defects evolve into in‐plane vacancies and topological defects.^[^ ^8^ ^]^ Further graphitization at the higher temperatures (1800–3000 °C) promotes the growth of graphitic grains and the restoration of conjugated sp^2^ structure,^[^ ^9^ ^]^ which help to enhance the phonon diffusion and electronic transport in the final films obtained.^[^ ^10^ ^]^

In the process described above, the repair efficiency of defects and the energy consumption during high‐temperature annealing have been a concern for a long time, which may restrict further commercial viability of highly ordered graphene films.^[^ ^11^ ^]^ Besides, the loss of carbon atoms upon heat treatment would deteriorate the yield of carbon and thus increase the cost of products eventually.^[^ ^12^ ^]^ To address this issue, numerous studies have been conducted to investigate the structural repair and optimize the graphitization protocol of graphene films.^[^ ^13^ ^]^ For example, Jin et al., found that reducing the graphitization temperature to ≈1900 °C could repair the structure of low‐defective graphene oxide made by thermal exfoliation, resulting in highly crystalline graphene platelets with a thickness of ≈0.56 nm, a lateral size of ≈153 nm and an electrical conductivity of ≈56 500 S·m^−1^.^[^ ^13a^ ^]^ Ghosh et al., performed the chemical reduction with hydrazine to remove the carbonyl oxygen‐containing groups, followed by thermal annealing at 1900 °C to obtain graphene films with an intensity ratio of D peak to G peak (I_D_/I_G_) of 0.15 by Raman spectroscopy and electrical conductivity of ≈16 000 S·m^−1^, higher than ≈14 000 S·m^−1^ for thermally reduced graphene.^[^ ^13b^ ^]^ Ishida et al., used ethanol as an extra carbon source to assist the repair of GO film at 1500 °C in bare N_2_ atmosphere to obtain the turbostratically stacked graphene with I_D_/I_G_ of 0.3, lower than I_D_/I_G_ of 0.6 from graphene made by reduction without carbon source.^[^ ^13c^ ^]^ In our previous research, ascorbic acid has been used as a reductant to promote the assembly of GO sheets and to simultaneously act as carbon source, resulting in an enhanced thermal conductivity of 1600 W·m^−1^·K^−1^ for an 80 µm‐thick film.^[^ ^14^ ^]^ Though the advances above, the repair of defects during the thermal annealing requires more precise control, with higher repair efficiency and lower carbon loss.

Recently, relatively perfect graphene has been found to be a good template to facilitate the efficient graphitization of polymer precursors. Cunning et al., used well‐crystallized single‐layer graphene (SLG) grown by chemical vapor deposition (CVD) as a “guiding agent” for the structural evolution of SU‐8 negative photoresist films at 2000 °C graphitization, resulting in a graphitized layer of SU‐8 parallel to the SLG.^[^ ^15^ ^]^ Huang et al., also reported a highly crystalline graphite film by graphitization of polyacrylonitrile (PAN) in a confined 2D space between GO sheets with a lateral size of 25–35 µm, exhibiting a high in‐plane thermal conductivity of 1282 W·m^−1^·K^−1^, which is 7.3 times higher than that of PAN‐derived film and surpasses that of pure graphene film (1201 W·m^−1^·K^−1^).^[^ ^16^ ^]^ The larger GO platelet with lateral size of 30–50 µm was also found to be helpful in reducing the mismatch between the 2D GO sheet and the 1D PAN, benefiting the graphitization and crystallization process.^[^ ^17^ ^]^ Although the exciting proceedings above, GO, as the most accessible precursor for graphene films, has rarely been attempted to be accompanied with SLG for the possible improved reduction and structural repair in the thermal annealing process. Specifically, the atomic scale and electronic level mechanism on how SLG improves the structural repair of defective graphene remains elusive.

In this study, we investigate the effect of SLG on the structural repair of reduced graphene oxide (rGO) by combining density functional theory (DFT) simulations and experiments. Simulations based on *ab‐initio* molecular dynamics (AIMD) show that the carbon skeleton of rGO is maintained and the loss of carbon atoms is reduced during thermal reduction when contact with the SLG. Further analysis of the electronic properties shows that the charge transfer from SLG to the defective graphene (DG), which is obtained after the complete removal of oxygen functional groups, promotes the adsorption of carbon residuals in the thermal treatment, and makes DG more stabilized and thus better repaired. Ex situ Raman spectroscopy studies confirm the lower density of defects (I_D_/I_G_ < 0.01) in rGO with SLG obtained at 2200 K, compared to that without SLG at same annealing temperature.

## 2 Results and Discussion

**Figure 1:**
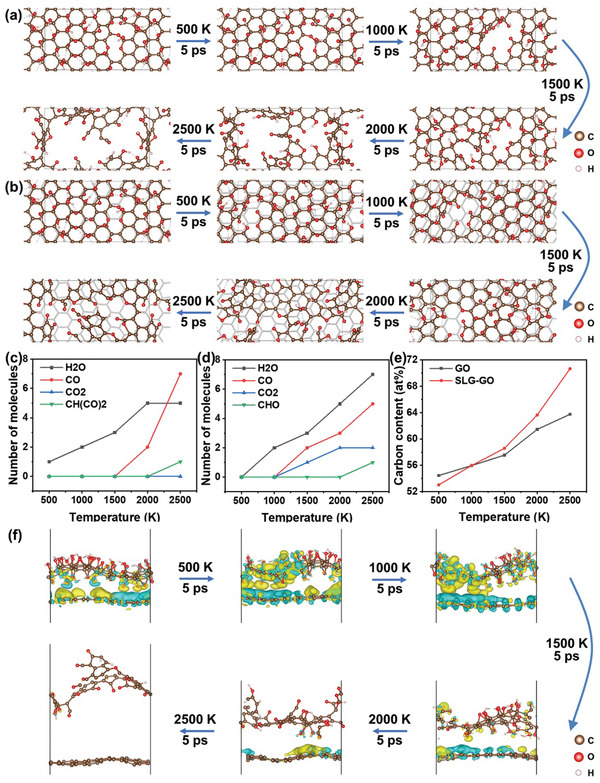
Structural evolution of a) bare GO and b) GO on SLG during the five‐step annealing, with 5 ps at constant temperature AIMD simulation for each step; Amount of released gas species from c) GO or d) GO on SLG in each annealing step; e) Carbon content of GO with or without SLG contact after each annealing; f) Side view of differential charge density of GO on SLG with optimized structure after each annealing step. The iso surface value is 0.0002 e bohr^−3^.

AIMD simulations were conducted to investigate the structural evolution of GO during high‐temperature annealing, by comparing two models: bare GO and GO on SLG. The five‐step annealing simulations were performed under the canonical ensemble (NVT), with temperatures at 500, 1000, 1500, 2000, and 2500 K in each step. A total time of 5 ps AIMD simulation was performed for each annealing step, with a time‐step of 1 fs. **Figure** **1**a shows the structural evolution of a bare GO during annealing. We can see that the in‐plane defects in bare rGO (GO is reduced upon heating) gradually enlarge as the temperature increases during annealing, due to the loss of carbon atoms in the reduction process of GO. The carbon skeleton undergoes fragmentation after annealing at 2500 K, with the edges saturated with oxygen residual groups. In contrast, the introduction of SLG under GO maintains the carbon skeleton, with smaller in‐plane vacancy defects in rGO, after the same annealing steps. Thus, the expansion of defects is slowed due to the presence of SLG, as shown in Figure 1b. The number of gas molecules generated from bare GO or GO on SLG is shown in Figure 1c,d, respectively. We can see that H_2_O molecules begin to release during annealing at 500 K for the bare GO but have been postponed to 1000 K for GO on SLG, showing the improved structural stability of GO on SLG. CO and CO_2_ are simultaneously generated from two samples for annealing temperatures above 1000 K, but GO on SLG tends to release CO_2_ rather than CO. Upon annealing at higher temperatures like 2500 K, more complex carbon‐containing radicals, e.g., CHO and CH(CO)_2_, are generated from both samples, indicating the weakened effect of SLG at so high temperatures. Correspondingly, the carbon content increases with the annealing temperature, but GO on SLG shows the higher carbon content for the annealing beyond 1500 K, as shown in Figure 1e. The differential charge density shown in Figure 1f indicates that an effective charge transfer occurs from SLG to rGO for the annealing at temperatures below 2500 K, potentially accounting for the enhanced stability of the carbon skeleton of rGO on SLG.^[^ ^6^ ^]^ At the same time, we can see that, for the annealing performed above 1000 K, the interlayer spacing between rGO and SLG gradually increases with the annealing temperature, due to the wrinkling of rGO, showing a diminishing interlayer interaction with reduced charge transfer strength. This partially explains the weakening effect of SLG on the repair of GO at high temperatures. The reaction paths for the release of CO, CO_2_, or H_2_O are explored by the Nudged Elastic Band (NEB) method, as shown in Figure S1 (Supporting Information). Combing the energy barrier (Figure S2, Supporting Information) and crystal orbital Hamilton population (COHP) (Figure S3, Supporting Information) analysis, these results all indicate that the presence of the SLG makes the desorption of CO harder and tends to release more content of H_2_O and CO_2_, which thus increase the reduction (deoxygenation) efficiency while preventing the loss of carbon atoms.

**Figure 2:**
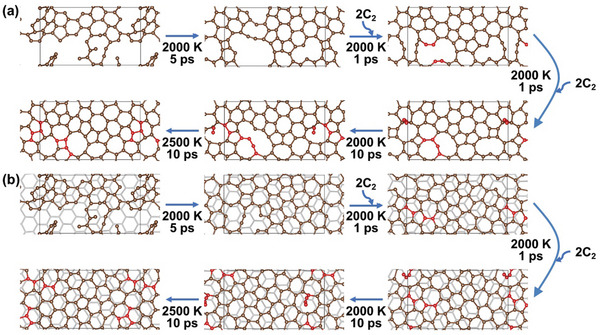
Structural restoration of DG a) and DG on SLG b) with feeding carbon source at constant temperatures of 2000 or 2500 K. Carbon dimers (red atoms) are introduced as extra carbon source in the annealing.

The repair of the carbon skeleton in graphitization was further studied by AIMD simulations starting from a model of defective graphene (DG). The bare DG is constructed by heating GO on SLG at 2000 K for 5 ps, as shown in Figure 1b, followed by the removal of residual oxygen and hydrogen atoms. **Figure** **2**a shows the AIMD simulation results for the repair of bare DG, by feeding with an additional carbon source, e.g., carbon dimers,^[^ ^18^ ^]^ in the part of annealing at 2000 K. We can see that at the beginning of the repair process at 2000 K, the adsorption of carbon dimer leads to the formation of carbon chains, as shown by Figure 2a after annealing at 2000 K for 6 ps. After the final annealing at 2500 K for 10 ps, most of the defects in bare DG are significantly restored, but a certain amount of high‐energy point defects, e.g., non‐hexagonal rings, remain. With the presence of SLG under the DG, as shown in Figure 2b, the repair is more efficient, evidenced by the faster reconstruction of the carbon skeleton and the absence of linear carbon chains. The obtained structure contains less point defects in the DG on SLG, compared to the bare DG. Thus, the presence of SLG can significantly benefit the repairing quality of DG during annealing, with the supplement of an additional carbon source.

**Figure 3:**
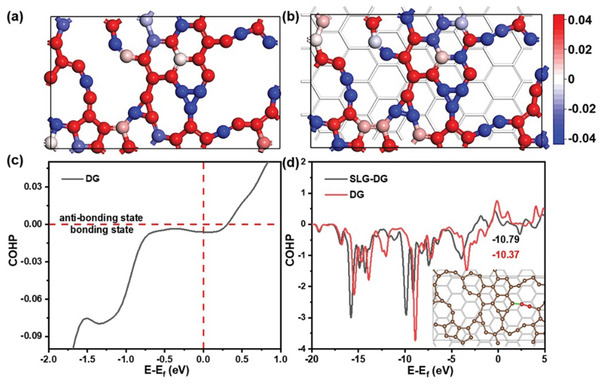
Bader charges analysis of a) bare DG and b) DG on SLG; c) COHP of all carbon‐carbon bonds inside DG. (d) COHP between DG and adsorbed dimer. The inset in d) shows the geometrical structure of DG as brown balls, the adsorbed dimer as red balls, and the bonds for COHP evaluation as green sticks.

To investigate the effect of SLG in the electronic aspect, the static calculation was performed with DG or DG on the SLG structure. The DG structure was obtained using the same method mentioned above. The SLG on the DG structure is obtained by placing the SLG under an optimized DG model, followed by a full optimization, as shown in Figure S4 (Supporting Information). Based on Bader charge analysis, the net charge of each carbon atom in DG or DG on SLG is shown in **Figure** **3**a,b, and also listed in Table S1 (Supporting Information). From the comparison, we can see that the DG is more negatively charged with the presence of SLG, showing a charge transfer of 3.58 × 10^−4^ e^−^ Å^−2^ from SLG to DG. In addition, the COHP analysis of all carbon bonds in DG at the Fermi level shows a bonding state, indicating that the negative charge transfer from SLG would lead to the occupation of bonding states, thus strengthening the carbon bonds, as shown in Figure 3c. That is, the presence of SLG has stabilized the carbon skeleton. On the other hand, we find that carbon dimers can be more effectively adsorbed on the model of DG on SLG, as shown in Figure S5a–d (Supporting Information). The analysis of COHP in Figure 3d and Figure S6 (Supporting Information) indicates that the carbon–carbon bonds between DG and dimer or the carbon‐carbon bonds inside the carbon dimer are both strengthened for DG on SLG. The higher absolute value of COHP integrated into the Fermi level (10.79 eV for DG on SLG vs 10.37 eV for isolated DG) suggests the higher bonding strength in DG on SLG. More analysis of COHP (Figure S5e,f, Supporting Information) suggests that the carbon‐carbon bonds in the DG is also strengthened with the presence of SLG, indicating the role of SLG on the structural preservation of DG at high temperatures.

Experiments have been conducted to prepare HOPG/rGO/SLG/GO to verify the effect of SLG during the thermal reduction of GO, as shown in the schematic diagram in Figure S7 (Supporting Information). The morphology of GO on SiO_2_/Si or mica substrates, and of GO/SLG or rGO/SLG/GO on predrilled HOPG has been characterized by SEM and AFM as shown in Figure S8a–e (Supporting Information). As shown by TEM and the electron diffraction pattern of SLG after annealing at 1000 °C (Figure S8f, Supporting Information), the annealed SLG largely maintains the crystal structure with hexagonal electron diffraction spots of graphene.

**Figure 4:**
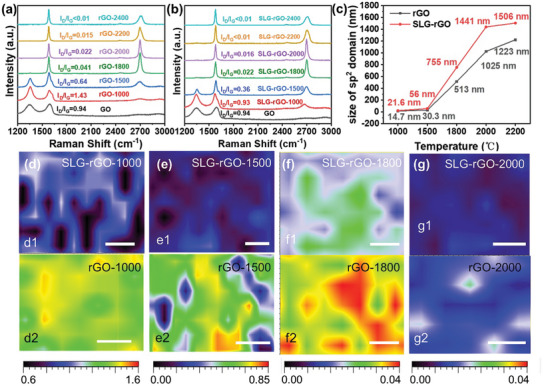
Raman spectra of a) rGO‐*x* and b) SLG‐rGO‐*x* at an excitation wavelength of 532 nm, where *x* denotes the annealing temperature; c) Size of sp^2^ domains as a function of the annealing temperature. d–k) Confocal Raman mapping of I_D_/I_G_ for samples of SLG‐rGO‐*x* and rGO‐*x*. Scale bars in the mapping images are 5 µm.

Hereafter, the GO region covered or uncovered by SLG in the same HOPG/rGO/SLG/GO sample is labeled as rGO‐*x* or SLG‐rGO‐*x* with *x* indicating the annealing temperature. **Figure** **4**a shows the Raman spectra of rGO‐*x* obtained at various annealing temperatures, with I_D_/I_G_ ratio marked. We can see that I_D_/I_G_ ratio maintains a value of 0.64 even after annealing at 1500 °C. In comparison, the I_D_/I_G_ ratio of SLG‐rGO (Figure 4b) starts to decrease at 1000 °C and is significantly reduced to 0.36 after annealing at 1500 °C, indicating the role of SLG in reducing defects in rGO. The appearance of second‐order two phonon mode located near 2700 cm^−1^ (2D band) can be fitted to symmetric Lorentzian–Gaussian peaks at 1800 °C and the single peak became prominent at 2000 °C, indicating the weakening of interlayer coupling in rGO. The evolution of the 2D peak is consistent with the reported functionalized graphene, which was obtained by thermally exfoliating graphite oxide.^[^ ^13a^ ^]^ The grain size (La) of sp^2^ region can be estimated by using I_D_/I_G_ with the equation: La = 2.4×10^−10^ × λ^4^ × (I_D_/I_G_)^−1^,^[^ ^19^ ^]^ where λ is the wavelength of laser of 532 nm. From Figure 4c we can see that when the temperature rises to ≈2000 °C, the grain size of SLG‐rGO has reached ≈1.4 µm. As the annealing temperature increases to 2200 °C, the increase of grain size becomes slow, suggesting a saturated grain growth. The near disappearance of the D peak brings challenges for accurate fitting, which occurs in graphitized rGO film at 2400 °C. The I_D_/I_G_ mapping across an area of ≈20 µm, as presented in Figure 4d–g, shows the SLG‐rGO has an overall lower defect ratio compared with rGO for the annealing temperature of 1000, 1500, and 1800 °C, while at 2000 °C, the difference is small due to the total diminishing of D band in both regions.

**Figure 5:**
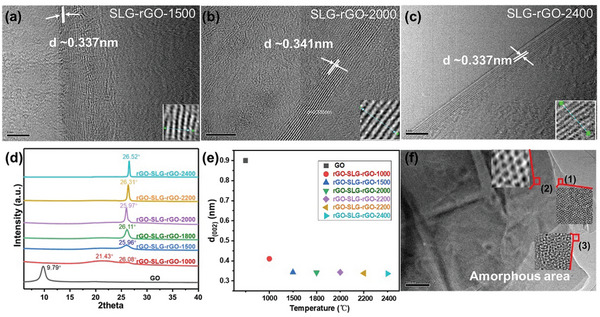
HR‐TEM images of a) SLG‐rGO‐1500, b) SLG‐rGO‐2000, and c) SLG‐rGO‐2400; all scale bars are 5 nm; d) XRD patterns of GO and multilayered films, which are composed of stacking rGO and SLG layers, starting from rGO layer, consisting of a total number of 15 layers of SLG and 16 layers of rGO, abbreviated as “rGO‐SLG‐rGO‐*x*”, where *x* denotes the annealing temperature; e) (002) interlayer spacing as a function of the heating temperature; f) Typical TEM image of SLG‐rGO‐2400 and the different region in amplified images in the insets, in which (1) (2) (3) are different regions framed in red in SLG‐rGO‐2400; the scale bar is 200 nm.

HRTEM and XRD have been used to track the structural evolution of SLG‐rGO films during the thermal annealing and the crystallization of SLG‐rGO films obtained at different annealing temperatures, as shown in **Figure** **5**. The HRTEM image in Figure 5a shows the clear graphene layers with 0.337 nm interlayer spacing in SLG‐rGO‐1500, suggesting the efficient repair of the conjugated structure in rGO. When the temperature increases to 2000 °C, the lamellae crystallinity of rGO significantly improves, forming continuous polycrystalline layers with a slightly increased lattice spacing to 0.341 nm (Figure 5b), in agreement with the observation of weaker interlayer coupling between layers, and consistent to the presence of a sharp single 2D peak in Raman spectra in Figure 4b. The higher annealing temperature, e.g., 2400 °C, transforms the diffraction patterns of rGO from polycrystalline rings to bright diffraction spots, as shown in Figure S9a–c (Supporting Information), indicating an improved crystallinity and stacking between layers, as also shown in HRTEM images in Figure 5c. The XRD spectra in Figure 5d show the broad band observed in SLG‐rGO‐1500, corresponding to the presence of curved edges observed in Figure 5a. The disappearance of the diffraction peak at ≈21° for SLG‐rGO‐1500 suggests the role of SLG in guiding the crystallization of rGO and reducing the fraction of the amorphous phase. With the increase in temperature, the (002) peak becomes sharper and the interlayer spacing quickly decreases, till values ≈0.33–0.34 nm for 2400 °C, as shown in Figure 5e. By analyzing the various stacking modes between layers in different magnified areas in the SLG‐rGO‐2400 sample (Figure 5f), we can see that most of the stacked layers are stacked in order, with a small amount of amorphous structures in some local areas, indicating the structure repair of the graphene layer is imperfect even after treatment at 2400 °C. The rGO‐SLG‐rGO‐2800 film, consisting of 15 layers of SLG (Figure S10, Supporting Information) and annealed at 2800 °C was analyzed to evaluate the effect of SLG in thick films. The average electrical conductivity of ≈8 µm‐thick rGO‐SLG‐rGO‐2800 film is 17 064.187 ± 784.921 S·cm^−1^, higher than 12 803.163 ± 1312.480 S·cm^−1^ for ≈7 µm‐thick film made by the same procedures yet without SLG.

## 3 Conclusion

Our results offer a route for understanding the structure evolution of carbon in the thermal repair of rGO at high temperatures. The combination of experimental and theoretical findings elucidates the impact of SLG on GO reduction, which maintains the carbon framework yet promotes the defect repair and growth of sp^2^ domains during graphitization. Our theoretical analysis highlights that the charge transfer from SLG to graphene stabilizes the defective graphene and enhances the carbon source adsorption, thus facilitating the production of higher‐quality graphene. Our work provides insight into the role of interactions between SLG and GO during the thermal graphitization process, thus enabling more efficient production of GO‐based high‐quality graphene films, thereby benefiting the potential applications that require high thermal and electrical conductivities.

## 4 Experimental Section

### Computational Methods

All calculations were performed using the DFT method implemented in the Vienna Ab‐initio Simulation Package (VASP).^[^ ^20^ ^]^ The Perdew—Burke–Ernzerhof (PBE) ^[^ ^21^ ^]^ exchange‐correlation functional with generalized gradient approximation (GGA) ^[^ ^22^ ^]^ was adopted. The long‐range van der Waals interactions were described by the DFT‐D3 method ^[^ ^23^ ^]^ with Becke–Jonson damping (BJ‐D3).^[^ ^24^ ^]^ The basis set cutoff energy was set at 400 eV and the Brillouin zone was sampled with a 1 × 1 × 1 gamma‐centered K‐points grid for structural optimization. The COHP ^[^ ^25^ ^]^ was calculated with a 4 × 2 × 1 gamma‐centered K‐points grid. The self‐consistent field (SCF) calculation was conducted with an energy convergence criterion of 10^−4^ eV and a force tolerance of 0.01 eV Å^−1^.

The simulation cell has a lattice of 17.38 × 10.03 Å with a 20.00 Å vacuum layer for both GO and SLG‐GO models. To achieve the better reduction effect,^[^ ^26^ ^]^ the GO model was set to contain 10 epoxy groups, 20 hydroxyl groups, 1 ether group, and 1 carbonyl group, based on an oxygen content of ≈30%, i.e., a hydroxyl/epoxy ratio of 2. The model structure of defective graphene (DG) for the simulation of annealing treatment was obtained by removing all the hydrogen and oxygen atoms from rGO annealed at 2000 K. The model structure of DG for the static calculation of the adsorption of dimer was obtained by removing all the hydrogen and oxygen atoms from rGO annealed at 2500 K.

### Chemicals and Materials

For the experimental synthesis of GO, chemical expandable graphite (CEG, E196403) was purchased from Sigma–Aldrich. To support GO/SLG membranes, highly oriented pyrolytic graphite (HOPG, 99.8% purity) was obtained from Alfa–Aesar (item No.13382). The SLG grown by CVD on Cu foil, abbreviated as Cu/SLG, was obtained from the Ningbo Soft Carbon Electronic Technology Co., *Ltd*., China. Poly (methyl methacrylate) (PMMA) was purchased from Aldrich. Potassium permanganate (KMnO_4_), sulphuric acid (H_2_SO_4_, 98 wt.%), hydrogen peroxide (H_2_O_2_), chlorobenzene, and ammonium persulfate ((NH_4_)_2_S_2_O_8_, 99.5 wt.%) were obtained from Sinopharm Chemical Reagent Co., *Ltd*., China and used without further purification. Acetone and isopropanol (IPA) were obtained from Tianjin Chemical Reagent Co. China.

### Fabrication of PTFE/GO Film

Graphite oxide was synthesized using a modified Hummers’ method (KMnO_4_/CEG = 3:1) without water‐enhanced oxidation, as previously described.^[^ ^27^ ^]^ The resulting graphite oxide was dialyzed to remove residual salts/acids and dispersed in water using sonication to obtain a brown GO suspension. The GO suspension was then subjected to centrifugation sequentially at 3000 and 6000 rpm each for 10 min to remove un‐exfoliated particles. The obtained GO suspension was then diluted to a concentration of ≈0.1 mg mL^−1^ and vacuum filtrated on a hydrophilic polytetrafluoroethylene (PTFE, 0.45 µm pore size) membrane for the preparation of a ≈50 nm‐thick GO film. The PTFE/GO hybrid film was then dried in an oven at 60 °C overnight.

### Wet Transfer of SLG Onto PTFE/GO

The SLG was transferred onto the PTFE/GO hybrid film using the wet transfer method, as previously reported.^[^ ^28^ ^]^ To perform the transfer, the Cu/SLG was cut into 1.5 cm × 1.5 cm squares, and one side of Cu/SLG was protected with PMMA (0.5 m PMMA/chlorobenzene solution by spin‐coating, followed by baking at 50 °C for 1 h). The PMMA‐protected Cu/SLG was then floated on 0.5 m (NH_4_)_2_S_2_O_8_ etchant for 3 h at 40 °C to remove Cu, and cleaned with deionized water five times. The resulting sample is named as PTFE/GO/SLG/PMMA.

### Fabrication of HOPG/rGO/SLG/GO

After immersing PTFE/GO/SLG/PMMA in ethanol for 10 min, a free‐standing GO/SLG/PMMA thin film was detached from the PTFE support and transferred onto HOPG for the high‐temperature treatment. To prevent the interference of the HOPG substrate in the Raman testing, the HOPG were pre drilled to obtain holes, leading to porous HOPG. The GO/SLG/PMMA on the porous HOPG was dried in ambient conditions. PMMA was first softened and detached from SLG by heating at ≈85 °C for 2 h, then was completely removed by immersing in a 3:1 acetone/isopropanol solution for 4 h, followed by rinsing with deionized water and drying in a vacuum. The obtained HOPG/GO/SLG was annealed at 400 °C for 30 min to remove residue PMMA to obtain a sample of HOPG/rGO/SLG (note GO was reduced to rGO after annealing at 400 °C). Finally, a diluted GO dispersion (<0.1 mg mL^−1^) was deposited onto the SLG side of the HOPG/rGO/SLG sample, resulting in a HOPG/rGO/SLG/GO sample after drying.

### Thermal Annealing of HOPG/rGO/SLG/GO

The HOPG/rGO/SLG/GO stacking film was subjected to a thermal treatment in a furnace (LF furnace series, Centorr Vacuum Industries company, USA) at temperatures ranging from 1000 to 2400 °C for 5 min at Ar atmosphere. The treated samples were abbreviated as “SLG‐rGO‐x”, where x denotes the annealing temperature. SLG was also directly transferred onto the surface of freshly cleaved HOPG instead of porous HOPG for atomic force microscopy (AFM). The same annealing process at temperatures ranging from 1000 to 2400 °C as described above was used for the HOPG/SLG sample.

### Multilayered Stacking of GO/SLG/GO

By repeating the transfer of SLG 15 times on PTFE/GO, PTFE/GO/SLG/GO/SLG/GO… multiple layers were prepared. The removal of the PTFE membrane was achieved by the same method mentioned above. These films were further annealed using a two‐step graphitization at 2000 and 2800 °C for 2 h each, followed by pressing under a pressure of ≈75 MPa to obtain highly densified graphene films consisting of 15 layers of SLG (abbreviated as “rGO‐SLG‐rGO‐x”, where x denotes the annealing temperature), as previously described in this work.^[^ ^27^ ^]^

### Characterizations

Field‐emission scanning electron microscopy (FESEM, FEI, Apreo, US) was utilized to determine the size and distribution of GO. The thickness of the GO/rGO platelets and GO films was measured by AFM (Nanoscope III MultiMode, Germany). High‐resolution transmission electron microscopy (HRTEM, JEOL‐2100F, Japan) was employed to analyze the microstructure of SLG‐deposited rGO sheets. Raman spectra (LabRAM, RM3000, Renishaw, UK, 532 nm laser) were obtained to investigate the microstructure of the final treated samples. The crystallinity of the continuous laminated rGO‐SLG‐rGO‐*x* films was evaluated using X‐ray diffraction (XRD) with Cu Kα radiation (λ = 1.5418 Å, tube voltage 40 kV, tube current 30 mA, Rigaku SmartLab, Japan). The electrical conductivity of rGO‐SLG‐rGO‐2800 film was measured via a standard four‐probe method using a Hall Effect testing system (Keithley 2182A/6430, China).

## Conflict of Interest

The authors declare no conflict of interest.

## Supporting information



Supporting Information

## Data Availability

The data that support the findings of this study are available from the corresponding author upon reasonable request.
