# Supplementary material for: Structural Repair of Reduced Graphene Oxide Promoted by Single‐Layer Graphene
Source: Adv Sci (Weinh). 2024 Dec 30;12(7):2410088. doi: 10.1002/advs.202410088 (PMC11831445; doi:10.1002/advs.202410088)
Supplement: Supplementary file 1 — Supporting Information [file ADVS-12-2410088-s001.docx]

**Supplemental Information**

**Structural Repair of Reduced Graphene Oxide Promoted by Single-Layer Graphene**

Minghao Guo, Hong Yuan, Kun Ni^*^, Chuanren Ye, Fei Pan, Juan Xiong and Yanwu Zhu*

Hefei National Research Center for Physical Sciences at the Microscale, & Department of Materials Science and Engineering, School of Chemistry and Materials Science, & Key Laboratory of Precision and Intelligent Chemistry, University of Science and Technology of China, Hefei, Anhui 230026, China

Corresponding authors: [nikun@ustc.edu.cn](mailto:nikun@ustc.edu.cn) (K. Ni); [zhuyanwu@ustc.edu.cn](mailto:zhuyanwu@ustc.edu.cn) (Y. Zhu)

**Supplementary figures**

**
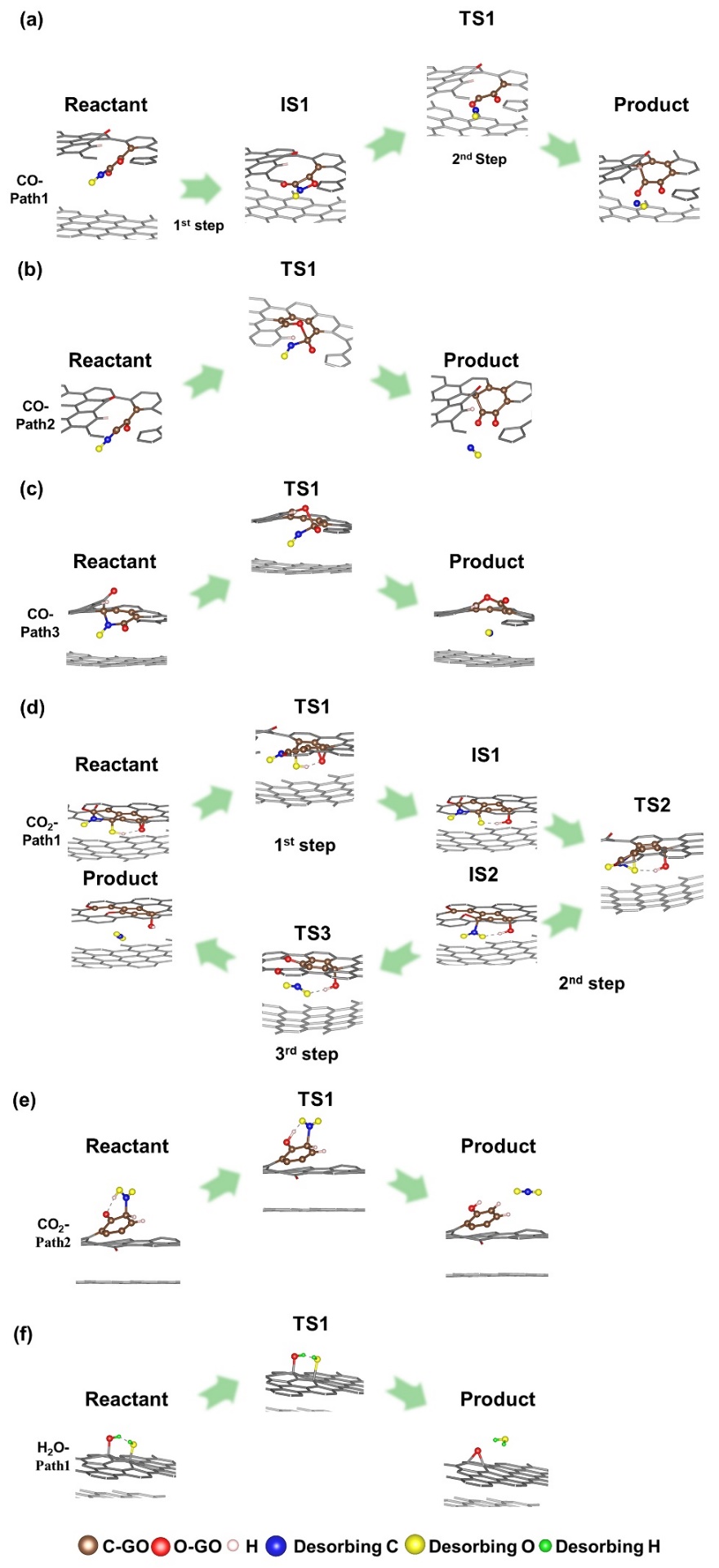
**

**Figure S1.** Reaction pathway for the reaction of (a) CO-Path1, (b) CO-Path2, (c) CO-Path3, (d) CO_2_-Path1, (e) CO_2_-Path2 and (f) H_2_O-Path1. The brown, red, white, blue, yellow and green spheres indicate carbon and oxygen atoms in GO on SLG, hydrogen atoms, carbon, oxygen and hydrogen atoms in the gas products, respectively.


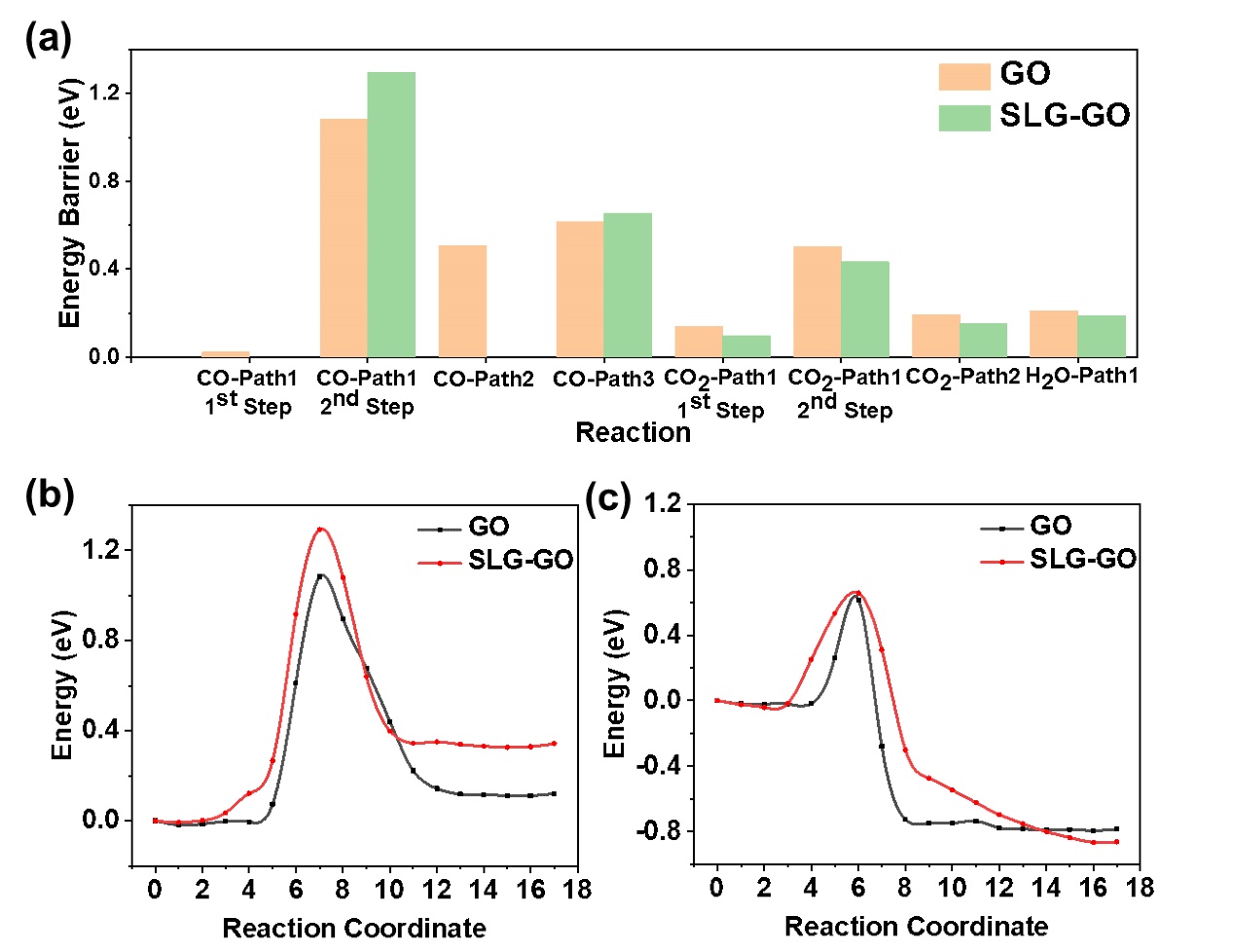


**Figure S2.** (a) Energy barrier comparison for the reactions in the formation of CO, CO_2_ and H_2_O in Figure S1(a); (b) Reaction pathway for the 2^nd^ step in the reaction of CO-Path1; (c) Reaction pathway for the reaction of CO-Path3. The presence of SLG increases the energy barriers for CO release, but decreases those of CO_2_ and H_2_O release, compared to the situation for bare GO.


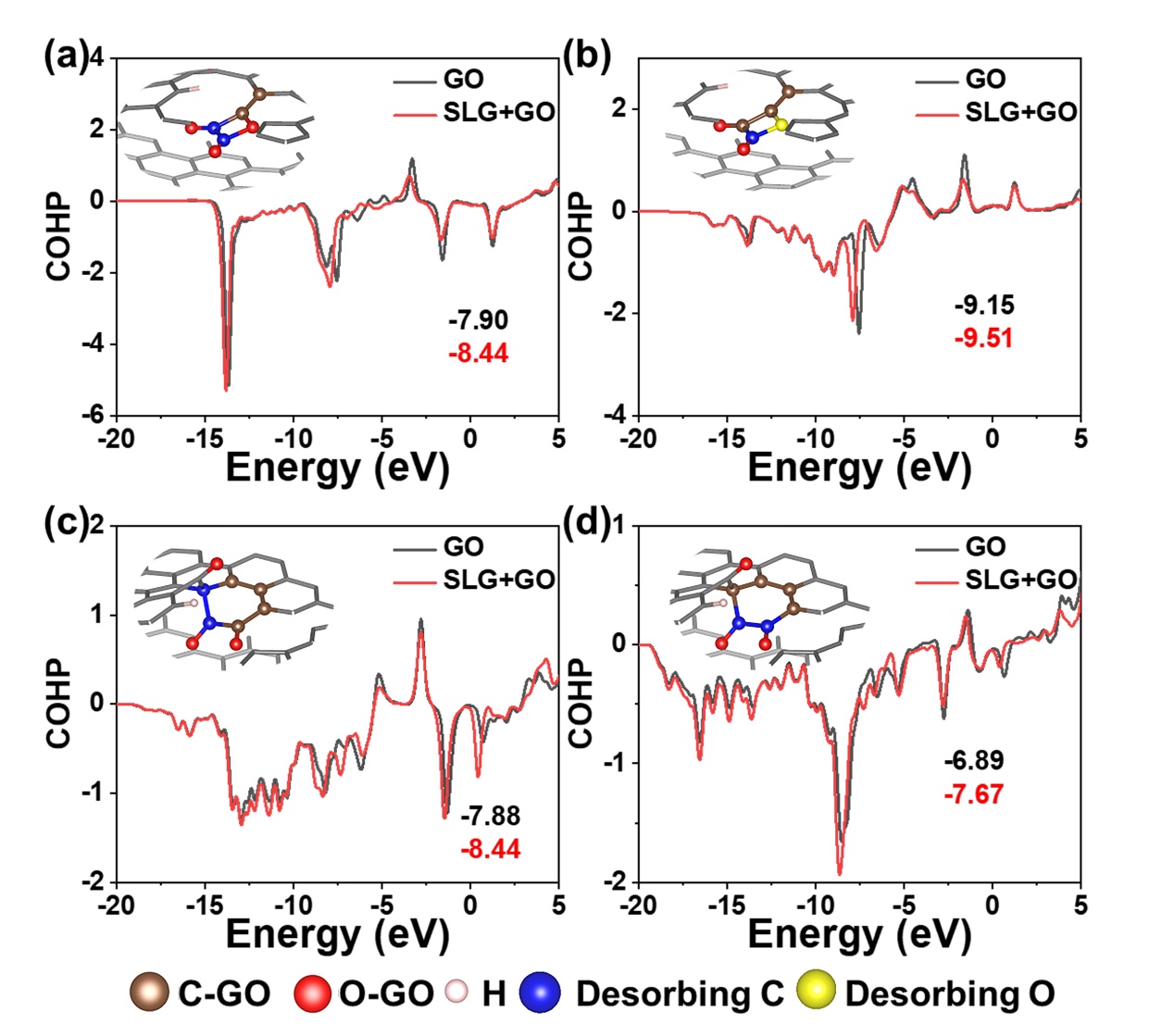


**Figure S3.** COHP between the desorbing atom and the adsorption sites (a)-(b) in CO-Path1 and (c)-(d) in CO-Path3, which releases a CO molecule. The brown, red, white spheres indicate carbon, oxygen, hydrogen atoms in GO, and the blue, yellow spheres indicate the desorbing carbon, oxygen atoms, respectively. The calculated COHP pairs are between blue C atoms in (a), (c) and (d), and between blue C atom and yellow O atom in (b).


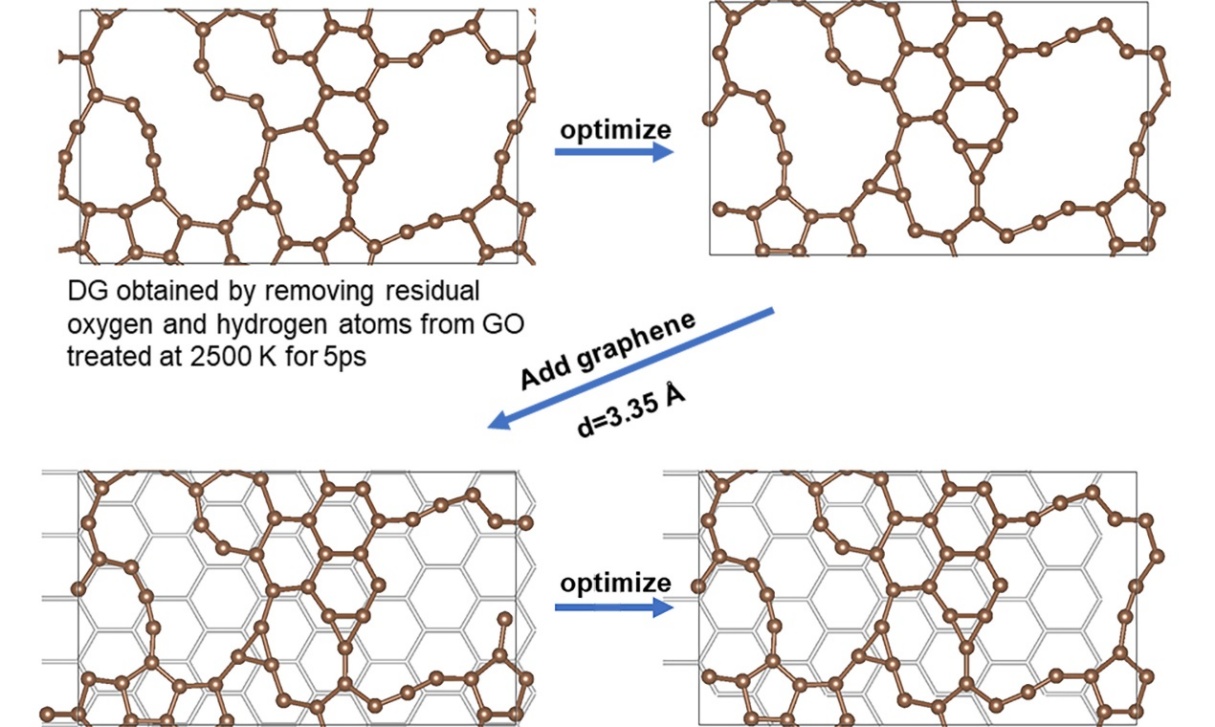


**Figure S4.** Optimization procedure to obtain SLG-DG model by adding a SLG layer under the DG layer, in order to maximumly maintain the DG structure for further comparation.


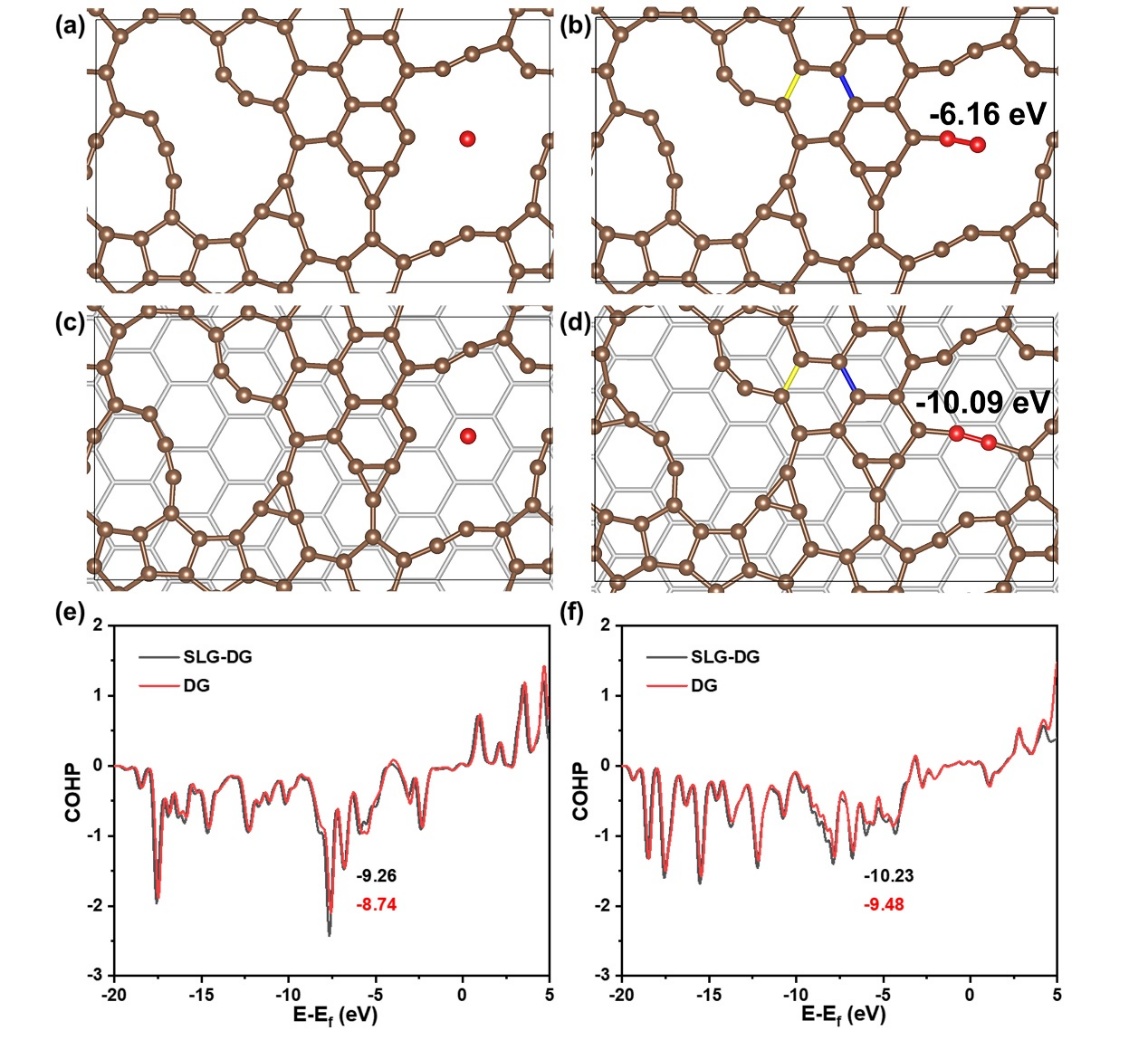


**Figure S5.** Dimer adsorption on DG before optimization(a) and after optimization(b) or DG with SLG before optimization(c) and after optimization(d); The labeled value in (b) and (d) is the adsorption energy of the dimer. COHP analysis of carbon-carbon bond between six-seven membered ring(e), which is labeled in yellow, or six-six membered ring(f), which is labeled in blue, in the defective graphene in (b) and (d).


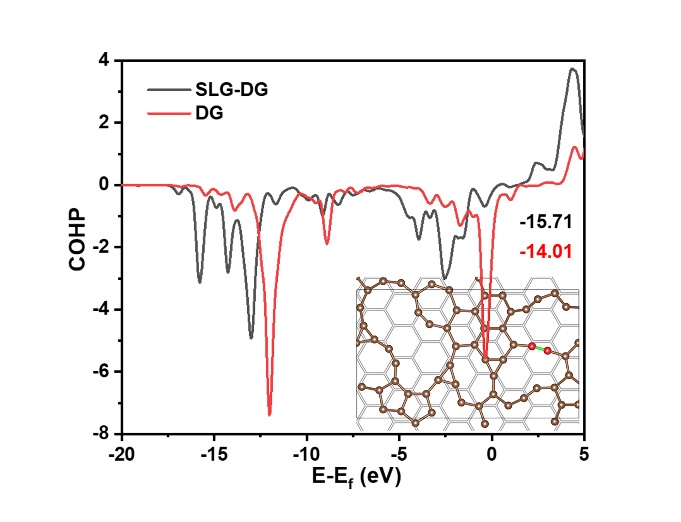


**Figure S6.** COHP analysis of carbon-carbon bonds inside the dimer, which is adsorbed on defective graphene.


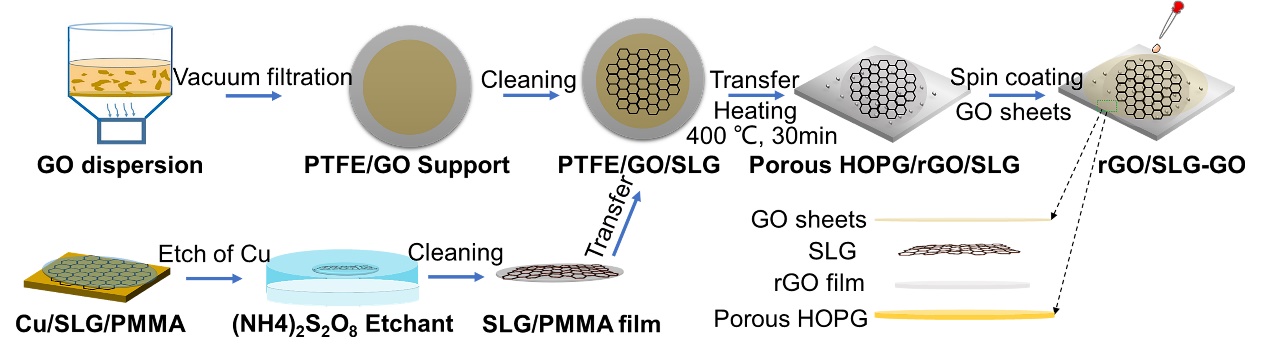


**Figure S7.** Schematic of the rGO/SLG-GO preparation procedure, based on wet transfer method.

**
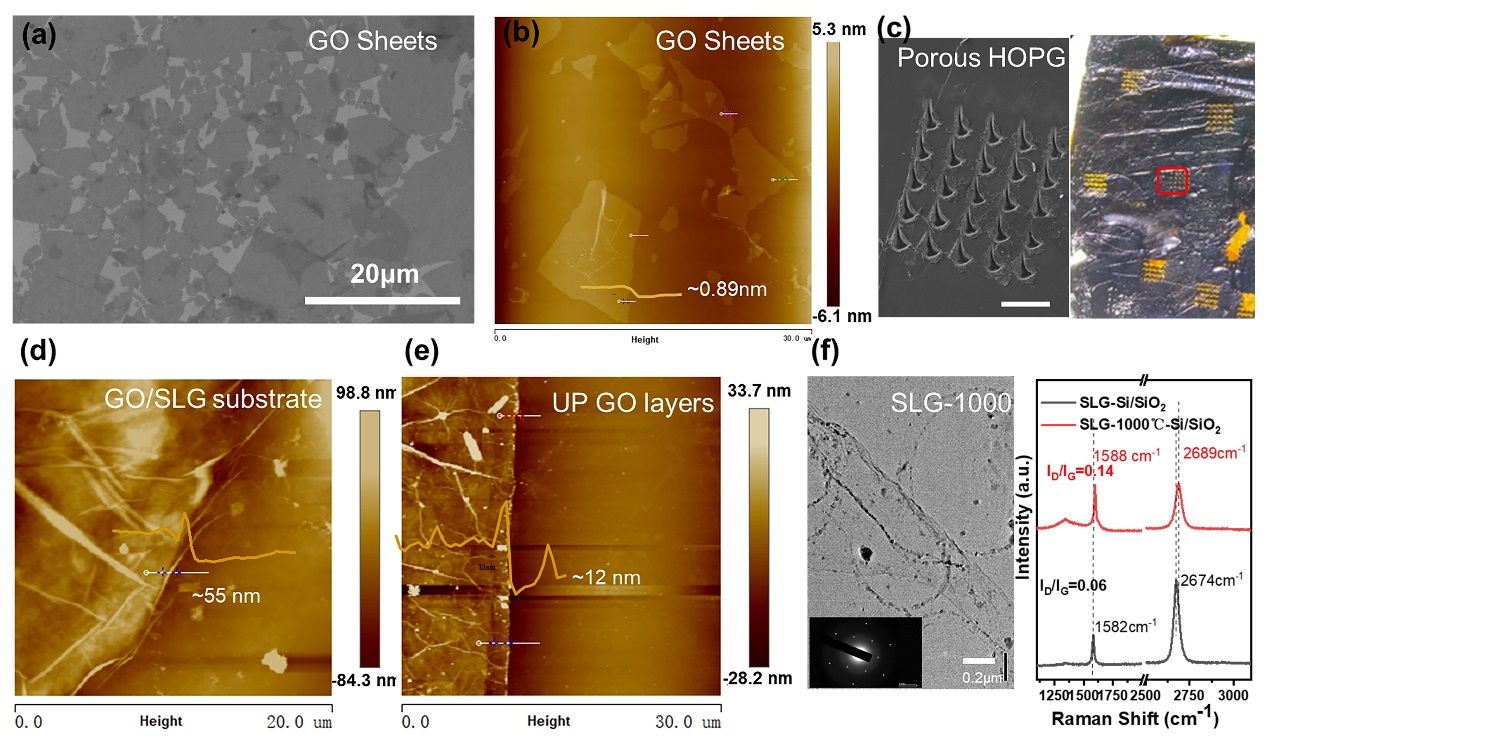
**

**Figure S8.** (a) SEM images of GO flakes casted on a SiO_2_/Si substrate, fabricated from KMnO_4_/CEG ratio of 3:1; (b) AFM image and line profile of GO sheets deposited on mica surface; (c) the SEM image of the porous HOPG substrate (left) and GO film on the porous HOPG substrate (right), which is transferred by wet method. (d) AFM image showing the thickness of the GO/SLG film substrate transferred;(e) the thickness of up GO layer dripped onto the rGO/SLG substrate from AFM image. (f) Typical TEM image of carbonization of SLG/PMMA at 1000 °C and its Raman spectra; the inset shows the selective electron diffraction of SLG-1000.

**
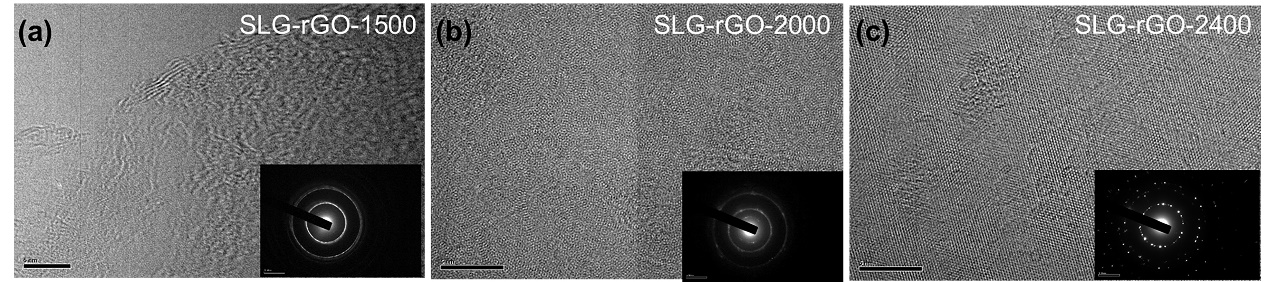
**

**Figure S9.** HR-TEM images and the local diffraction patterns of (a) SLG-rGO-1500, (b) SLG-rGO-2000 and (c) SLG-rGO-2400; The scalebar is 5 nm.

**
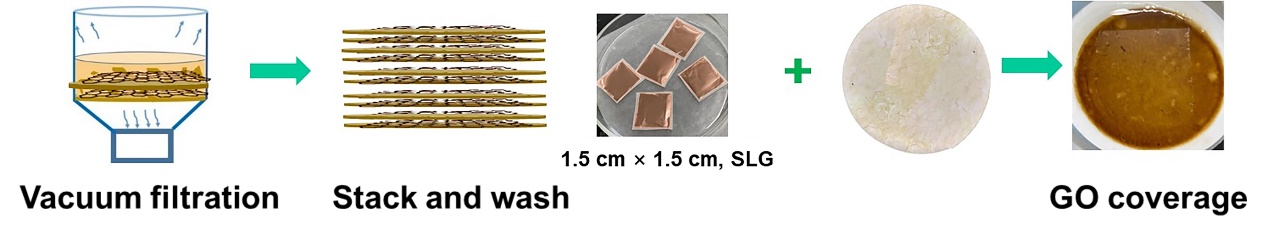
**

**Figure S10.** Schematic of the preparation of the multilayer stacked GO/SLG/GO graphene film sample, including assembly and transfer.

**Supplementary tables**

**Table S1.** Number of atoms with specific net charge in DG or DG on SLG based on Bader analysis.

| Color | net Charge | In DG | In DG on SLG |
| --- | --- | --- | --- |
| Blue | <-0.04 | 11 | 17 |
| white | -0.04<&<0.04 | 13 | 13 |
| red | >0.04 | 27 | 21 |
